# Looking at the Nudibranch Family Myrrhinidae (Gastropoda, Heterobranchia) from a Mitochondrial ‘2D Folding Structure’ Point of View

**DOI:** 10.3390/life11060583

**Published:** 2021-06-18

**Authors:** Giulia Furfaro, Paolo Mariottini

**Affiliations:** 1 Department of Biological and Environmental Sciences and Technologies—DiSTeBA, University of Salento, I-73100 Lecce, Italy; 2 Department of Science, University of Roma Tre, I-00146 Rome, Italy; paolo.mariottini@uniroma3.it

**Keywords:** 2D RNA-Barcoding, molecular morphology, Nudibranchia, *Dondice*

## Abstract

Integrative taxonomy is an evolving field of multidisciplinary studies often utilised to elucidate phylogenetic reconstructions that were poorly understood in the past. The systematics of many taxa have been resolved by combining data from different research approaches, i.e., molecular, ecological, behavioural, morphological and chemical. Regarding molecular analysis, there is currently a search for new genetic markers that could be diagnostic at different taxonomic levels and that can be added to the canonical ones. In marine Heterobranchia, the most widely used mitochondrial markers, COI and 16S, are usually analysed by comparing the primary sequence. The 16S rRNA molecule can be folded into a 2D secondary structure that has been poorly exploited in the past study of heterobranchs, despite 2D molecular analyses being sources of possible diagnostic characters. Comparison of the results from the phylogenetic analyses of a concatenated (the nuclear H3 and the mitochondrial COI and 16S markers) dataset (including 30 species belonging to eight accepted genera) and from the 2D folding structure analyses of the 16S rRNA from the type species of the genera investigated demonstrated the diagnostic power of this RNA molecule to reveal the systematics of four genera belonging to the family Myrrhinidae (Gastropoda, Heterobranchia). The “molecular morphological” approach to the 16S rRNA revealed to be a powerful tool to delimit at both species and genus taxonomic levels and to be a useful way of recovering information that is usually lost in phylogenetic analyses. While the validity of the genera *Godiva*, *Hermissenda* and *Phyllodesmium* are confirmed, a new genus is necessary and introduced for *Dondice banyulensis*, *Nemesis* gen. nov. and the monospecific genus *Nanuca* is here synonymised with *Dondice*, with *Nanuca sebastiani* transferred into *Dondice* as *Dondice sebastiani* comb. nov.

## 1. Introduction

The use of molecular techniques to investigate the evolutionary history of animal groups and to define monophyletic lineages expanded exponentially in the last few decades, becoming one of the essential steps to accomplish a good integrative taxonomy. To increase the robustness of the obtained results, the integration of different kinds of informative characters has led to an exhaustive search for characters that could be diagnostic at different taxonomic levels. Integrative taxonomy applied to marine Heterobranchia is proving to be a fruitful field of study. In fact, in the last few years, many papers have been published, which included information derived from several biological points of view, including morphological, molecular, chemical, ecological and behavioural characters [1,2,3,4,5,6,7]. Regarding the molecular approach, there is a continuous search in the genome for new informative DNA coding and non-coding regions, consequentially resulting in molecules such as proteins and RNA [7,8,9]. Mitochondrial DNA (mtDNA) is currently, in the greatest number of cases, the most powerful genome being exploited to shed light on lower taxonomic levels, such as species and genera, and it remains the most used DNA molecule to delimit species and investigate cryptic diversity and recent speciation events [7,10,11,12]. In fact, animal mitochondrial DNA is characterised by a high copy number, largely maternal inheritance, lack of recombination, and displays a higher mutation rate than the nuclear DNA. It is affected by some constraints including retention of ancestral polymorphism, male-biased gene flow, selection on any mtDNA nucleotide, introgression following hybridisation and paralogy resulting from transfer of mtDNA gene copies to the nucleus [13,14,15,16]. To avoid these pitfalls, nuclear markers are usually added to the molecular analyses. This approach has been applied to marine Heterobranchia (Mollusca, Gastropoda) whose evolutionary history, when studied at family level, is reconstructed using mainly three molecular markers, the two mitochondrial genes, part of Cytochrome oxidase subunit I (COI) and part of the ribosomal subunit 16S (16S), as well as the nuclear gene histone 3 (H3) which is well known to be poorly or not quite informative at lower taxonomic levels [7,17,18]. By means of these markers, the systematics of several heterobranchs families has been clarified and erroneous outcomes derived from previous morphological studies have been resolved and corrected [2,3,5].

However, the potential of some aspects of the known mitochondrial genome are still incompletely exploited today. These include ribosomal RNA genes, which are transcribed into rRNA molecules able to fold into secondary/tertiary structures that are necessary for correct mitoribosome assembly [19]. The primary sequence of some regions of the mitochondrial rRNA is hypervariable and difficult to align among related species. This rRNA hypervariability occurs in the mitochondrial ribosomal 16S RNA (16S rRNA), used as a classical marker, which folds into different domains and stem-loops. When alignments based on the primary sequences are not easy to achieve, it is a common and suggested practice to remove these portions to stabilise the phylogenetic signal (there are specific programs that help to cut these hypervariable regions, for example the GBlocks program [20,21]). The removal of these unreliable alignment regions from the dataset is promoted by several influential works aimed to demonstrate the improvement of statistical support of the phylogenetic analyses when randomness in sequence alignments is cut out [21,22,23]. However, this practice means that, unavoidably, very informative regions are often not taken into consideration.

In the last few decades, the comparison of the secondary (2D) structure information of RNA molecules, such as the nuclear ribosomal RNA internal transcribed spacer 2 (ITS2 rRNA) in molluscs (bivalves), has been revealed as a promising approach in both phylogenetic reconstruction and species diagnosis [24,25,26,27]. In particular, an important character that has proved very useful are Compensatory Base Changes, CBCs, that are defined as two mutations that occur in a paired region of a primary RNA transcript so that pairing itself is maintained (e.g., G-C mutates to A-U) [28]. On the contrary, information derived from the folded structure of the 16S rRNA has not been utilised in molluscan groups, except for two works by Furfaro et al. [18,29], who analysed this barcoding marker in a couple of sympatric sibling sea slugs (gastropods). These authors reported 2D structural diversity of the 16S rRNA, mainly utilising the highly variable L7 and L13 stem-loops [30], demonstrating that secondary RNA structure information is a valuable additional diagnostic tool for integrative taxonomy and species delimitation [18,29].

In this study, we have extended the approach of 2D RNA-barcoding, investigating systematics at a taxonomic level higher than the species level. In particular, it was used as a case study in a polyphyletic clade [31] composed of four genera ranked in the family Myrrhinidae Bergh, 1905: *Dondice* Marcus Er. 1958; *Godiva* Macnae, 1954; *Hermissenda* Bergh, 1879 and *Phyllodesmium* Ehrenberg, 1831. To date, these genera include four accepted species for *Dondice* and *Godiva* respectively, three for *Hermissenda* and 27 accepted species for *Phyllodesmium*, which is the Myrrhinid genus that is richest in species. These Myrrhinid genera were previously considered part of the polyphyletic family Facelinidae, but were recently moved to the family Myrrhinidae by Martynov et al. [32], based on phylogenetic evidence from a systematic study focused on other families (i.e., Tergipedidae). To date, phylogenetic relationships among these genera and the included species are still incompletely resolved. In fact, there was evidence that the genus *Dondice* as traditionally conceived is not monophyletic and needs an in-depth study based on a wider dataset [31,32]. The family Myrrhinidae includes charismatic species, which is distributed worldwide and interesting from different points of view. *Hermissenda crassicornis* (Eschscholtz, 1831) is a model organism used in various research fields, such as neurology, ecology, ethology, pharmacology and toxicology [33,34,35,36,37,38,39]. Species belonging to the genus *Phyllodesmium* are a group of highly specialised nudibranchs with sophisticated species-specific mechanisms of mimicry with external body features indistinguishable from their cnidarian hosts and are animal models for several studies on chemistry and cell biology or focused on the mechanisms of the symbiotic relationship with the zooxanthellae living in their digestive gland cells [40,41,42]. This family was chosen as a case study to investigate the capability of the 2D folding structure analyses to give useful insight on the systematics of this animal group by looking at alternative morphological, yet molecular, features. This choice was due to the peculiarity of the genera involved, their unresolved phylogenetic histories and a unique ecological defensive strategy shown by most of the species involved (i.e., the capability to autotomise their cerata if disturbed [31]).

Suitable in-group and out-group species were selected, and the definitive dataset defined by being able to describe, with statistically supported analyses, the evolutionary history among representatives of the family Myrrhinidae. Particular attention was given to the inclusion of all the type species of the genera considered in the present study. Phylogenetic analyses were performed, based on an enlarged dataset that includes additional related genera, to investigate the evolutionary history of this interesting group of sea slugs. According to the phylogenetic analysis carried out, the 16S rRNA folding structures of all the type species of the genera belonging to the family Myrrhinidae (*Dondice occidentalis* (Engel, 1925); *Hermissenda opalescens* (J. G. Cooper, 1863); *Godiva quadricolor* (Barnard, 1927); *Phyllodesmium hyalinum* Ehrenberg, 1831] plus *Nanuca sebastiani* Er. Marcus, 1957, currently ascribed to the Family Facelinidae Bergh, 1889 and of *Aeolidiella alderi* (Cocks, 1852), *A. sanguinea* (Norman, 1877) (Aeolidiidae Gray, 1827), *Babakina anadoni* (Ortea, 1979), *B. indopacifica* Gosliner, Gonzalez-Duarte & Cervera, 2007 (Babakinidae Roller, 1973) and *Dicata odhneri* Schmekel, 1967 (Facelinidae Bergh, 1889)) were examined for comparison. After a stem-loop structure analysis of the 16S rRNA molecule, the specific and highly variable L7 stem-loop was chosen as the most divergent and informative for this group and was revealed to be diagnostic to unambiguously discriminate different 16S rRNA structures. This approach, based on the description of the “molecular morphology” of this very variable region of the mitochondrial 16S rRNA [18], can be considered as an additional tool for species delimitation and integrative taxonomy in Heterobranchia and more generally in marine molluscs. Considering all these issues, the aims of the present work were to: (1) provide an updated phylogenetic framework for the genera of the family Myrrhinidae; (2) describe for the first time the consensus 2D secondary structure of the genus *Dondice*; (3) investigate the diagnostic power of the 16S 2D molecular morphology as an additional tool useful for integrative taxonomy and for shedding light on the particularly intricate history of the family Myrrhinidae; (4) introduce *Nemesis* gen. nov. as a new genus of the family Myrrhinidae; (5) rename the taxon *Nanuca sebastiani* to *Dondice sebastiani* comb. nov.

## 2. Materials and Methods

### 2.1. Phylogenetic Analyses

The dataset consisted of 47 sequences, retrieved from GenBank, belonging to 30 species, including the out-group (Table 1). The mitochondrial markers COI and 16S and the nuclear marker H3 are commonly used in nudibranch phylogenetics (e.g., [6,17,43]), the mitochondrial ones being highly variable and informative at a shallow level of divergence, and the nuclear one having a slow rate of mutation, and thus, being more suitable to detect deep divergences in the tree, such as those with a distant out-group.

The selected sequences (Table 1) were aligned using the Muscle algorithm implemented in MEGA 6.0 [44]. The Gblocks 0.91b web server [20,21] was used to remove the hyper-divergent regions of the 16S rDNA alignment, using the less stringent parameter setting. For each gene alignment the best evolutionary model was selected by JModelTest 0.1 [45] according to the Bayesian Information Criterion (BIC).

Downstream phylogenetic analyses were performed on the single 16S dataset and on the concatenated and partitioned dataset. Phylogenetic analyses were performed using Bayesian Inference (BI) and Maximum Likelihood (ML) methods. *Duvaucelia striata* Haefelfinger, 1963 was used as the out-group in BI and ML phylogenetic analyses based on previous tests aimed to find the best in-group and out-group. BI analyses were carried out with MrBayes 3.2.6 [46], implementing the models selected by JModel Test for each gene partition. We ran four Markov-chains of five million generations each, sampled every 1000 generations. Consensus trees were calculated on trees sampled after a burn-in of 25%. MCMC chain convergence was verified by average standard deviation of split frequencies values below 0.006 and confirmed in Tracer 1.7 [47]. Maximum Likelihood analyses were performed in raxmlGUI 1.5b2 [48], a graphical front end for RAxML 8.2.1 [49], with 100 independent ML searches and 1000 bootstrap replicates (command “-f b”), applying the general time-reversible model with a gamma model of rate heterogeneity (GTRGAMMA), with individual gene partitions.

### 2.2. RNA Secondary Structure Modelling and Compensatory Base Changes (CBCs)

Partial 16S rRNA (sequence region from L7 to L13 stem-loops) secondary structure was obtained using the program “The Mfold web server” [50,51]. The best-supported folding models were predicted combining a thermodynamic approach [52] with a close study of the paired conserved regions and the identification of CBCs and semi-CBCs.

### 2.3. Morphological Analyses

To provide additional morphological data that were missing in the original description of *Dondice banyulensis*, anatomical analyses of the radula and jaws from two individuals of *D. banyulensis* were carried out. Buccal masses were removed and dissolved in a Proteinase K solution for the extraction of the chitinous structures. Radulae and jaws were rinsed in water, dried and mounted for examination by optical microscopy. The buccal structures were then mounted and gold coated in an Emitech K550 unit for SEM analysis. To obtain high resolution pictures, SEM images at different magnification levels were performed using the JSM-6480LV Scanning Electron Microscope (JEOL Ltd., Tokyo, Japan) at the Laboratorio di Microscopia Elettronica, Department of Mathematics and Physics, University of Salento. Analyses of the reproductive system of *D. banyulensis* were carried out by anatomical dissection under a stereomicroscope.

## 3. Results

### 3.1. Phylogenetic Analyses

**Figure 1:**
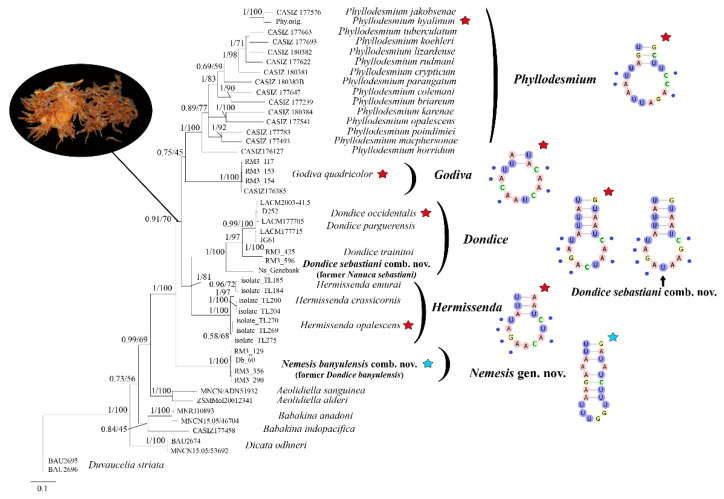
Bayesian phylogenetic tree based on the concatenated dataset (H3, 16S, COI). Bayesian posterior probability (**left**) and Bootstrap (**right**) values are indicated at each node. Red stars highlight the type species of the genera analysed and the relative 16S 2D L7 structures. The blue stars refer to the type species of the *Nemesis* gen. nov. and its 16S rRNA 2D L7 structure. In the black oval at the top left, *G. quadricolor* is shown as well as the autotomy of its cerata as the defensive strategy characteristic and common to the monophyletic group which includes *Dondice*, *Godiva*, *Hermissenda* and *Phyllodesmium*.

The definitive dataset consisted of 134 sequences from 47 specimens belonging to 30 different species, including the out-group (Figure 1, Table 1). The single 16S dataset consisted of 350 nucleotides, while the final concatenated alignment was 1232 nucleotides long. The best evolutionary models selected by JModelTest 0.1 according to the Bayesian Information Criterion (BIC) were: TIM1 + I + G, HKY + I + G and TIM2ef + G for the COI, 16S and H3 alignments, respectively. Results from Bayesian and Maximum Likelihood analyses yielded congruent topologies (Figure 1) both grouping all the *Phyllodesmium* species in a single and strongly supported (BI = 1; ML = 100) monophyletic clade, sister to *Godiva quadricolor* (BI = 1; ML = 100) with a low statistical support (BI = 75; ML = 45). These two clades are sister to another monophyletic group (BI = 1; ML = 81), which includes one monophyletic clade (BI = 1; ML = 100) that grouped species such as *Dondice occidentalis* and *D. parguerensis* (BI = 0.99; ML = 100) and *D. trainitoi* (BI = 1; ML = 97) sister to *Nanuca sebastiani* (BI = 1; ML = 100), and another monophyletic clade, which includes all the species belonging to the genus *Hermissenda* (BI = 1; ML = 100). *Dondice banyulensis* is the sister to all this big monophyletic clade (BI = 0.91; ML = 70), with strong statistical support (BI = 1; ML = 100). The two *Aeolidiella* sister species (BI = 1; ML = 100) are sister to all the previously reported clades forming a large monophyletic group (BI = 0.99; ML = 69). The latter group is sister to *Babakina* spp. (BI = 73; ML = 56) with *Dicata odhneri* (BI = 1; ML = 100) basal to all the previously reported clades and with the out-group *Duvaucelia striata* as the basal sister group to all the mentioned species. BI and ML analyses of the single 16S dataset yielded congruent results but with low statistical support (Figure S1).

### 3.2. 16S RNA Primary and Secondary Structures Analysis

**Figure 2:**
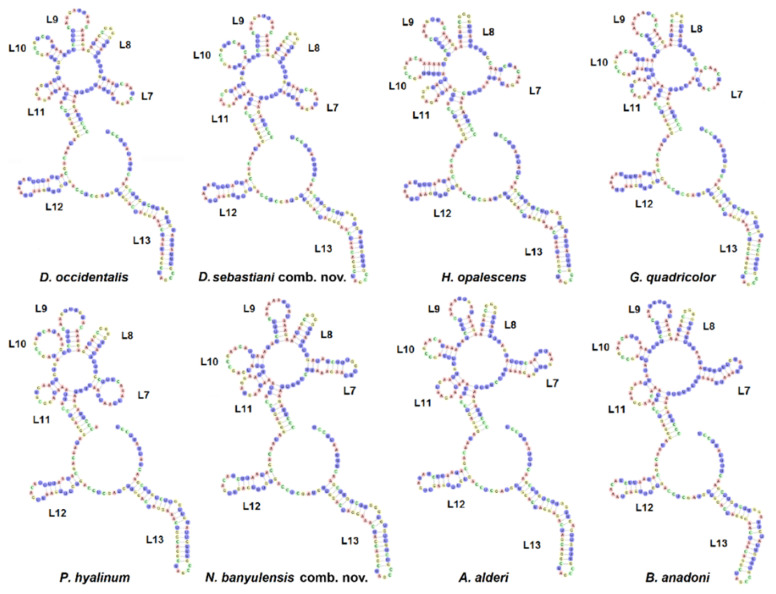
Folding models of the 16S rRNA V domain of the representative species analysed in the present work.

**Figure 3:**
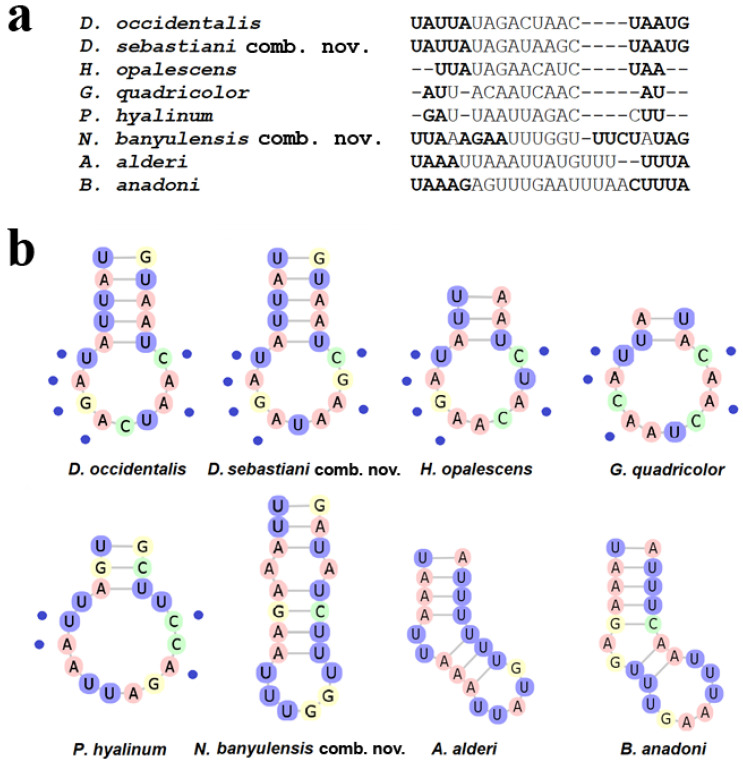
L7 stem-loops of the species here analysed. (**a**) Primary sequence alignment of the L7 stem-loops among the species analysed in the present work. (**b**) Blue circles indicate the conserved nucleotide positions in *Dondice*, *Nanuca, Hermissenda, Godiva* and *Phyllodesmium* genera.

The mitochondrial 16S rRNA multiple sequence alignments were performed simultaneously considering the secondary structure of each sequence to obtain the optimisation of the final alignment. The 16S rRNA single gene alignment consisted of 407 positions and 47 sequences, obtained from 30 species belonging to 9 accepted genera, including the out-group (Table 1). We have analysed the secondary structure (2D) of the 3′ half portion of the 16S rRNA gene, corresponding to the V domain and including the L7-L13 stem-loops [30,53] (Figure S3), in order to highlight the different diagnostic nucleotides characterising genus and species [54]. No variability in the folding 2D structures was detected within the same species. As expected, the V domain showed a high conservation of the primary sequence and global folding when compared among the species analysed (Table 1), which conforms to the canonical molluscan architecture [18,24,25,29,30,53]. Among the variable stem-loops, the L7 was the most divergent rRNA sequence containing diagnostic nucleotides and it was taken into consideration as an RNA barcoding region [18,29]. In Figure 2, the folding models of the 16S rRNA V domain are displayed of eight representative species belonging to seven genera. The L7 stem-loops analysed range from 14 (*G. quadricolor*) to 23 (*B. anadoni*) nucleotides (nt) in length and share a high homology of the sequence/2D structure among three *Dondice* spp. (i.e., *D. occidentalis, D. parguerensis* and *D. trainitoi*) and the monotypic *N. sebastiani* (Figure 3). As shown in Figure 3, the stem-loop is composed of 19 nucleotides in these taxa, the stem composed of five base pairs is identical, while the nine nt long loop shows six identical and three variable nucleotides. Unexpectedly, the L7 stem-loop of *Dondice banyulensis* revealed to be not correlated to the one of the congeneric species when considering both the primary sequence and 2D structure, exhibiting no significant folding homology (Figure 3). Moreover, in Figure 3 the L7 stem-loops of *H. opalescens*, *G. quadricolor*, *P. hyalinum*, *A. alderi* and *B. anadoni* are depicted for comparison. The secondary structure of the *H. opalescens* L7 stem-loop, chosen as an example of the genus *Hermissenda* since it is the type species of the genus, reveals a close homology in sequence/folding with the ones of *D. occidentalis* and *N. sebastiani*, which are the type species of *Dondice* and *Nanuca* genera, respectively; the nucleotide sequence of the shorter stem in particular is identical, while the eight nucleotides-long loop shares six identical nucleotides (Figure 3). Interestingly, two groups of nucleotide positions can be observed in the L7 stem-loop of the members grouped in the *Dondice* clade, which is formed by three taxa (Figure 3 and Figure S4): the first, variable and diagnostic for species identification and the second conserved and diagnostic at the genus level. Three diagnostic nucleotide substitutions separate *Dondice* spp. and the closely related *Nanuca* taxon, indicating that the latter monotypic genus is strongly related to *Dondice*. On the other hand, four nucleotide positions are conserved and shared in the clade which includes both genera *Dondice* and *Nanuca*. Taking into account the folding models of the L8-11 stem-loops, the 2D structures of these hairpins revealed a closer homology between *D. occidentalis* and *N. sebastiani* (Figure S3) than with *D. banyulensis*. In Figure S3, the L8-11 stem-loops are circled to show both the 2D structure and the nucleotides differences occurring among the three taxa. In particular, despite the dissimilar loop size of the L10 stem-loop, due to a single mismatch in the stem, *D. occidentalis* and *N. sebastiani* share a higher 2D folding homology than with *D. banyulensis.* Regarding the type species of the *Godiva* genus, the *G. quadricolor* L7 stem-loop is slightly divergent in sequence and 2D structure, but still exhibiting five identical nucleotides in its 10 nucleotides-long loop. The folding homology decreases analysing the 2D structures of the L7 stem-loop of *P. hyalinum*, a species type of the genus *Phyllodesmium* (four identical nucleotide in the 12 nt long stem). Two other examples of the L7 stem-loop refer to *A. alderi* and *B. anadoni*, which both show very divergent 2D structures.

To summarise, the L7 stem-loop of *D. banyulensis* revealed to be the more divergent 2D structure compared to the ones of *Dondice* spp., *N. sebastiani*, *H. opalescens*, *G. quadricolor* and *P. hyalinum*, exhibiting no significant folding homology (Figure 3 and Figure S4). According to both phylogenetic and 16S rRNA 2D structure analyses, the monotypic genus *Nanuca* must be synonymised with *Dondice*, and consequently, the taxon *Nanuca sebastiani* can be renamed to *Dondice sebastiani* comb. nov.

### 3.3. Morphological Analyses

**Figure 4:**
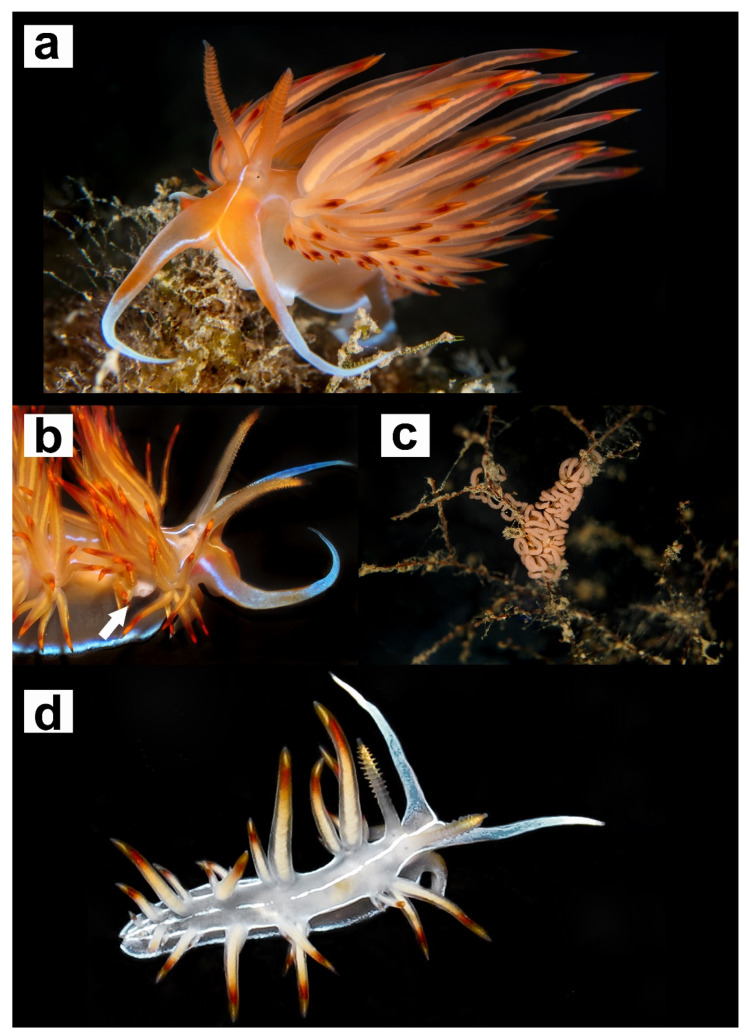
Images of individuals belonging to *Nemesis banyulensis* comb. nov. (original name *Dondice banyulensis*) (**a**,**b**,**d**) and the typical egg ribbon (**c**) of the species. (**a**) Adult specimen from the ‘Peschereccio’ wreck, Gallipoli, Apulia (Central Tyrrhenian Sea, Mediterranean Sea). (**b**) Particular of the anterior right portion of one specimen from ‘Asia’ wreck in Civitavecchia (Central Tyrrhenian Sea, Mediterranean Sea) and its reproductive openings highlighted with a white arrow. (**c**) The typical orange to pinkish egg ribbon lead on a hydroid colony in Sant’ Agostino, Civitavecchia (Central Tyrrhenian Sea, Mediterranean Sea). (**d**) Picture of a juvenile individual from Scilla in Calabria (South Tyrrhenian Sea, Mediterranean Sea) showing the difference in the chromatic pattern from young to adult individuals.

**Figure 5:**
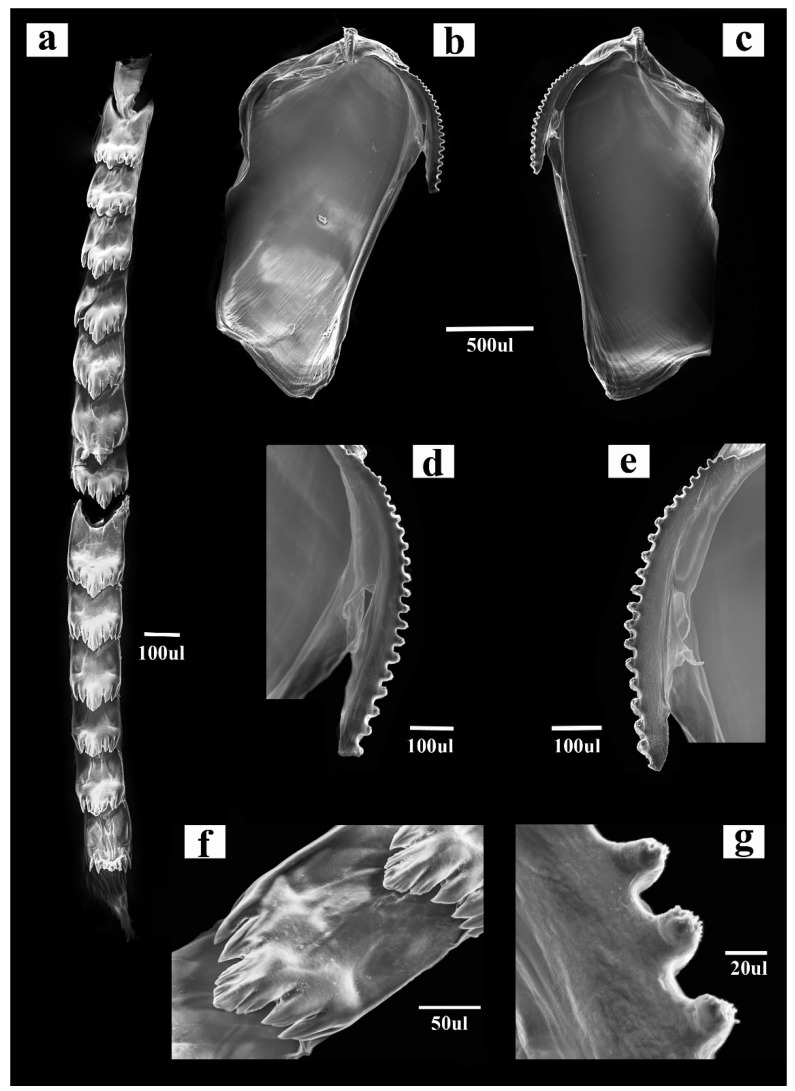
SEM images of the jaws and radula of *Nemesis banyulensis* comb. nov. (former *Dondice banyulensis*) (voucher RM3_290) (**a**), the entire radula of (**b**,**c**) both the left (**b**) and the right (**c**) jaws (**d**,**e**) particular of the masticatory boarders of the jaws. (**f**) Detail of the tooth of the radula with the cusp and the lateral denticles clearly visible. (**g**) The denticulated denticles of the masticatory border of the jaws at a high magnification level.

Morphological analyses of the buccal apparatus from two specimens (Vouchers RM3_290 and RM3_311) belonging to *Dondice banyulensis* (Figure 4) are shown in Figure 5. The high-resolution SEM pictures obtained showed that features of the jaws and radula are congruent with drawings reported in the original description [55]. The reproductive system was examined and the resulted drawing congruent with the one reported from the holotype (Figure S2).

## 4. Discussion

Based on these results, *Dondice banyulensis* clearly represents an independent lineage from the species of *Dondice* s.s. and deserves a genus on its own.

Taxonomy

Family Myrrhinidae Bergh, 1905

*Nemesis* gen. nov.

urn:lsid:zoobank.org:act:8F4E1E1D-6E8A-41CB-81FA-487A58829C65

Figure 4 and Figure 5 and Figure S1

Type species. *Dondice banyulensis* Portmann & Sandmeier, 1960

Etymology. The genus name was chosen to recall the Greek goddess *Nemesis* and her sense of justice to allocate the former *D. banyulensis* in the appropriate genus.

Diagnosis. Myrrhinid with rhinophores annulate and produced anterior foot corners. It has jaws with a single row of denticles that can be denticulated in their apical part. The central cusp of the radular tooth is not marked and is a little longer than lateral denticles. The large stomach, followed by a short smooth intestine, terminates with the anus, which is cleioproct and located inside the second group of cerata on the right side of the body. The long distal and proximal deferent ducts of the male portion of the reproductive system terminates with the unarmed penis. It is not able to autotomise the cerata when stressed by possible predators.

Species included. *Nemesis banyulensis* (Portmann & Sandmeier, 1960), comb. nov. (Original description in [55]; additional data in Figure 4 and Figure 5 and Figure S2).

Remarks. The new genus is diagnosed by a radular tooth characterised by the cuspid that is slightly marked and a little longer than lateral denticles compared to *Dondice* s.s., *Godiva* and *Phyllodesmium* and by the more elongated distal and proximal male deferent ducts of the reproductive system compared to *Dondice* s.s., *Godiva* and *Phyllodesmium*.

Mitochondrial DNA is, to date, the most powerful DNA molecule able to discriminate at species and genus taxonomic levels and is necessary to carry out any integrative systematic study [15,56,57,58,59,60]. In fact, even if today a lot of work is being done to find markers that are alternative to and independent from mitochondrial DNA, such as nuclear markers, it is undeniable that mitochondrial DNA is currently the most informative at lower taxonomic levels. Evidence for this is that nowadays there are two markers that are commonly used in most of the nudibranch systematic studies: the mitochondrial COI and the 16S. While waiting to find useful nuclear alternatives for the study of the evolutionary relationships that occurred between highly related groups, it may be useful to improve and optimise the study of the currently available markers, especially if these have characteristics that are potentially very useful; however, little is known of these characteristics in nudibranchs. One of these features is the ability of the 16S rRNA to fold into a 2D structure, which is mandatory for correct mitoribosome assembly [19]. In the mitochondrial 16S rRNA molecule, the V domain includes the L7-L13 stem-loops [30,53] that can be used to search for diagnostic nucleotides [54]. The 2D structure analysis using the 16S rRNA in molluscs has revealed to be capable to discriminate a couple of sympatric sibling sea slugs [18,29]. In particular, the stem-loop L9 nucleotide analyses confirmed the separation of the species *Diaphorodoris alba* Portmann & Sandmeier, 1960 and *D. luteocincta* (M. Sars, 1870), revealing some diagnostic nucleotides producing a 2D structural diversity [18]. Moreover, the L7 and L13 stem-loops resulted to be diagnostic in separating the two sibling Mediterranean species *Caloria elegans* (Alder and Hancock, 1845) and *Facelina quatrefagesi* (Vayssière, 1888) [29].

The case of the family Myrrhinidae is particularly intriguing due to the importance of the charismatic species included and because the genera belonging to this family were the object of recent integrative taxonomic studies, which, however, could not resolve important aspect of their systematics [31,32]. The latter studies have highlighted the possible polyphyletic nature of the Myrrhinidae genera that invoked more in-depth studies to resolve open questions. For this reason, in the present work, a broad dataset, including more individuals and species, was investigated using another, until now poorly known, source of information: the 2D structure of the 16S rRNA marker. In particular, we analysed the L7 stem-loop folding models since this small region proved to be very variable (Figure 2 and Figure 3, Figures S3 and S4). Two groups of nucleotide positions can be observed in the L7 stem-loop of the members grouped in *Dondice* clade (Figure 1): the first group is more variable and was diagnostic for species identification, while the second one includes nucleotides shared by all the taxa of the clade, and for this reason, could be very useful as a source of landmarks able to delimit the genus (Figure S4). Differences in nucleotides usually occur within the loop region and not in the stem, since the latter is under base-pairing constraints, which is what happens in the L7 stem-loop of *Dondice* spp. where no CBCs nor semiCBCs have been observed. The ability showed by the 2D folding structures of the 16S, and, in particular, by its small L7 loop, to give useful information at both species and genus taxonomic levels is quite important and reported here for the first time also at the genus level. This consideration becomes even more evident if we consider that, to improve results from phylogenetic analyses and from other methods of species delimitation, we are forced to exclude the hypervariable and unreliable regions of the 16S marker from our dataset, as strongly suggested by authoritative works [21,22,23]. The possibility to recover such a small but very significative region that would be otherwise lost constitutes an opportunity that must be considered.

The results from the phylogenetic approach and 16S rRNA 2D structures analyses are congruent with each other and yield a similar scenario, demonstrating the power of integrating these two methods. According to the results obtained, it is proposed that the species *Nanuca sebastiani*, currently included in the family Facelinidae and in the monospecific genus *Nanuca*, should be included in the genus *Dondice* (and consequently, moved to the family Myrrhinidae) as *Dondice sebastiani* comb. nov., with the genus *Nanuca* regarded as a synonym of *Dondice*. On the contrary, *Dondice banyulensis*, historically included in the genus *Dondice*, must be removed from the latter genus and ascribed as the type species of the new genus *Nemesis* gen. nov. here described, as *Nemesis banyulensis* comb. nov. Some very important ecological and morphological characters strongly support *Nemesis* gen. nov. within the Myrrhinidae. First of all, there is an ethological character (which indeed reflects the structural physiological difference) that appeared in the family after *Nemesis banyulensis* speciated, which became the synapomorphy of all the other apical genera (i.e., *Hermissenda*, *Dondice*, *Godiva* and *Phyllodesmium*): the ability to autotomise single or groups of cerata when under a predator’s attack or when in stressed conditions (showed in the black circle in Figure 1). This behaviour was a fundamental turning point in the evolution of these clades, as it constituted a new defensive strategy that was effective in increasing the survival skills of these shell-less sea slugs; in situ and laboratory observations confirm that *Nemesis banyulensis* does not perform cerata autotomy [31]. From a morphological and anatomical points of view, there are two other features that support the validity of *Nemesis* gen. nov.: (i) in the new genus, the radula lacks a prominent cusp, which is, instead, characteristic of the related genera (i.e., *Dondice* (see [31], Figure 4F), *Hermissenda, Godiva* and *Phyllodesmium*}. The median cusp of the radular tooth in *Nemesis* gen. nov. is not accentuated (Figure 5f); (ii) in *Nemesis* gen. nov., the distal and proximal male duct of the reproductive system is very long (Figure S2), which is typically different from other related genera (e.g., *Dondice* and *Godiva* see [31] Figure 5F). The results from morphological, ecological, molecular and 16S 2D structure analyses were congruent with each other, revealing the power of integrating evidence from several methods. In particular, the ‘molecular morphology’ approach revealed to be effective in this group of eolid nudibranchs, being able to give information at species and genera levels by looking at few diagnostic nucleotides. In fact, the diagnostic nucleotides associated to the 16S rRNA L7 stem-loop can be considered landmarks useful to separate at both genus and species levels in the family Myrrhinidae.

## 5. Conclusions

We have analysed the secondary structure of the 3′ half portion of the 16S rRNA gene in the representative species of five genera, i.e., *Dondice*, *Nanuca*, *Godiva*, *Hermissenda* and *Phyllodesmium*, searching for short variable regions displaying diagnostic nucleotides. Among the variable 16S rRNA stem-loops, the L7 resulted to be the most divergent and it was taken into consideration as a short 2D RNA barcoding region. The 2D folding analysis of this 19 nucleotides-long region produced the same phylogenetic relationship obtained with the Bayesian and Maximum Likelihood analysis on the concatenated H3, 16S and COI alignment (1232 nucleotides), pointing out the resolution power of the “molecular morphology” approach when integrated with the standard use of the primary sequence. We confirm the validity of the genera *Godiva*, *Hermissenda* and *Phyllodesmium* as traditionally conceived, while we propose to exclude *Dondice banyulensis* from the genus *Dondice* and to assign it as the type species of the new genus *Nemesis* gen. nov. Furthermore, the monospecific genus *Nanuca* is here synonymised with the *Dondice* genus, and consequently, the taxon *Nanuca sebastiani* is renamed to *Dondice sebastiani* comb. nov.

## Figures and Tables

**Table 1:** Species name, collection localities, Voucher IDs and sequence accession numbers (H3: Histone 3; 16S; COI: Cytochrome Oxidase subunit 1) of the specimens analysed.

SPECIES	LOCALITY	VOUCHER	H3	16S	COI
*Aeolidiella alderi* (Cocks, 1852)	Italy	ZSMMol20012341	HQ616795	HQ616766	HQ616729
*Aeolidiella sanguinea* (Norman, 1877)	France (Atlantic Ocean)	MNCN/ADN51932	JX087600	JX087538	JX087466
*Babakina anadoni* (Ortea, 1979)	Brazil	MNRJ10893	HQ616775	HQ616709	HQ616746
*Babakina anadoni* (Ortea, 1979)	Galicia, Spain	MNCN15.05/46704	HQ616796	HQ616730	HQ616767
*Babakina indopacifica* Gosliner, Gonzalez-Duarte & Cervera, 2007	Luzon, Batangas, Philippines	CASIZ177458	HM162587	HM162678	HM162754
*Dicata odhneri* Schmekel, 1967	Ballanera, Algesiras, Spain	BAU2674	LT596569	LT596549	LT596560
*Dicata odhneri* Schmekel, 1967	Andalusia, Spain	MNCN15.05/53692		HQ616739	HQ616773
*Dondice banyulensis* Portmann & Sandmeier, 1960	Djerba, Tunisia	RM3_129	LS483284	LS483274	LS483267
*Dondice banyulensis* Portmann & Sandmeier, 1960	Argentario, Tuscany, Italy	RM3_356	LS483285	LS483275	LS483268
*Dondice banyulensis* Portmann & Sandmeier, 1960	Sant’Agostino, Latium, Italy	RM3_290	LS483286	LS483276	LS483269
*Dondice banyulensis* Portmann & Sandmeier, 1960		Db_60		GQ403751	GQ403773
*Dondice occidentalis* (Engel, 1925)	Exuma, Bahamas	LACM177715	KC526529	KC526510	
*Dondice occidentalis* (Engel, 1925)		LACM2003-41.5	JQ699394	JQ699482	JQ699570
*Dondice occidentalis* (Engel, 1925)	Exuma, Bahamas	D252	KC526527	KC526518	
*Dondice occidentalis* (Engel, 1925)	Jamaica	JG61	KC526534	KC526512	
*Dondice parguerensis* Brandon & Cutress, 1985	La Parguera, Puerto Rico	LACM177705	KC526535	KC526520	
*Dondice trainitoi* Furfaro & Mariottini, 2020	Civitavecchia, Latium, Italy	RM3_425	LS483287	LS483277	LS483270
*Dondice trainitoi* Furfaro & Mariottini, 2020	Civitavecchia, Latium, Italy	RM3_596	LS483288	LS483278	LS483271
*Godiva quadricolor* (Barnard, 1927)	Sabaudia, Latium, Italy	RM3_117	LS483289	LS483279	MG546001
*Godiva quadricolor* (Barnard, 1927)	Sabaudia, Latium, Italy	RM3_153	LS483290	LS483280	MG546002
*Godiva quadricolor* (Barnard, 1927)	Sabaudia, Latium, Italy	RM3_154	LS483291	LS483281	MG546003
*Godiva quadricolor* (Barnard, 1927)	Knysna Lagoon, South Africa	CASIZ176385	HM162589	HM162680	HM162756
*Hermissenda opalescens*	Monterey Bay, CA, USA	isolate_TL270	KU950225	KU950130	KU950196
*Hermissenda opalescens*	Malibu, CA, USA	isolate_TL275	KU950224	KU950129	KU950195
*Hermissenda opalescens*	Long Beach, CA, USA	isolate_TL269	KU950222	KU950128	KU950193
*Hermissenda emurai*	Tateyama-Chiba, Japan	isolate_TL185	KU950215	KU950123	KU950186
*Hermissenda emurai*	Tateyama-Chiba, Japan	isolate_TL184	KU950214	KU950122	KU950185
*Hermissenda crassicornis*	Victoria, B.C., Canada	isolate_TL200	KU950210	KU950118	KU950174
*Hermissenda crassicornis*	Victoria, B.C., Canada	isolate_TL204	KU950212	KU950121	KU950178
*Nanuca sebastiani*			JQ699469	JQ699557	JQ699633
*Phyllodesmium briareum* (Bergh, 1896)	Batangas, Philippines	CASIZ 177239	HQ010460	HQ010528	HQ010492
*Phyllodesmium colemani* Rudman, 1991	Batangas, Philippines	CASIZ 177647	HQ010466	HQ010534	HQ010498
*Phyllodesmium crypticum* Rudman, 1981	Batangas, Philippines	CASIZ 180381	HQ010477	HQ010543	HQ010507
*Phyllodesmium horridum* (Macnae, 1954)	Cape Region, South Africa	CASIZ176127	HM162590	HM162681	HM162757
*Phyllodesmium hyalinum* Ehrenberg, 1831		Phy.orig.		GQ403756	GQ403778
*Phyllodesmium jakobsenae* Burghardt & Wägele, 2004	Batangas, Philippines	CASIZ 177576	HQ010456	HQ010524	HQ010489
*Phyllodesmium karenae* Moore & Gosliner, 2009	Batangas, Philippines	CASIZ 180384	HQ010478	HQ010544	HQ010508
*Phyllodesmium koehleri* Burghardt, Schrödl & Wägele, 2008	Batangas, Philippines	CASIZ 177693	HQ010462	HQ010530	HQ010494
*Phyllodesmium lizardensis* Burghardt, Schrödl & Wägele, 2008	Batangas, Philippines	CASIZ 180382	HQ010474	HQ010540	HQ010505
*Phyllodesmium macphersonae* (Burn, 1962)	Batangas, Philippines	CASIZ 177493	HQ010453	HQ010522	HQ010487
*Phyllodesmium opalescens* Rudman, 1991	Batangas, Philippines	CASIZ 177541	HQ010450	HQ010519	HQ010485
*Phyllodesmium parangatum* Ortiz & Gosliner, 2003	Batangas, Philippines	CASIZ 180383B	HQ010476	HQ010542	HQ010506
*Phyllodesmium poindimiei* (Risbec, 1928)	Batangas, Philippines	CASIZ 177783	HQ010463	HQ010531	HQ010495
*Phyllodesmium rudmani* Burghardt & Gosliner, 2006	Batangas, Philippines	CASIZ 177622	HQ010461	HQ010529	HQ010493
*Phyllodesmium tuberculatum* Moore & Gosliner, 2009	Batangas, Philippines	CASIZ 177663	HQ010465	HQ010533	HQ010497
*Duvaucelia striata* Haefelfinger, 1963	Giannutri Is., Tuscany, Italy	BAU2695	LT615407	LT596542	LT596540
*Duvaucelia striata* Haefelfinger, 1963	Formiche Is., Tuscany, Italy	BAU2696	LT615408	LT596543	LT596541

## Data Availability

Data available in a publicly accessible repository that does not issue DOIs. Publicly available datasets were analysed in this study. This data can be found here: [https://www.ncbi.nlm.nih.gov/].

## Supplementary Materials

The following are available online at https://www.mdpi.com/article/10.3390/life11060583/s1, Figure S1: Bayesian phylogenetic tree based on the single 16S dataset. Bayesian posterior probability (left) and Bootstrap (right) values are indicated at each node. Figure S2: Drawing of the reproductive system of *Nemesis banyulensis* comb. nov. (Voucher RM3_290) performed using optical microscope. Legend: amp = ampulla, bc = bursa copulatory, dd = deferent duct, fgm = female gland mass, md = male duct, p = penis, rs = *receptaculum seminis*, v = vagina. Figure S3: (a) Folding models of the 16S rRNA V domain of *D. occidentalis*, *D. sebastiani* comb. nov. and *N. banyulensis* comb. nov. analysed in the present work. The L7-11 stem-loops are boxed. (b) The variable L9-11 stem-loops are encircled to show differences occurring among the three taxa. Figure S4: L7 stem-loops of the species here analysed. (a) Primary sequence alignment of the L7 stem-loops among the *Dondice* spp. and *N. banyulensis* comb. nov. (b) Folding models of the L7 stem-loops. Blue circles indicate conserved nucleotide positions within the *Dondice* clade and red circles indicate diagnostic nucleotides.
