# Supplementary material for: Looking at the Nudibranch Family Myrrhinidae (Gastropoda, Heterobranchia) from a Mitochondrial ‘2D Folding Structure’ Point of View
Source: Life (Basel). 2021 Jun 18;11(6):583. doi: 10.3390/life11060583 (PMC8235141; doi:10.3390/life11060583)
Supplement: Supplementary file 1 [file life-11-00583-s001.zip › life-1205152-supplementary.pdf]

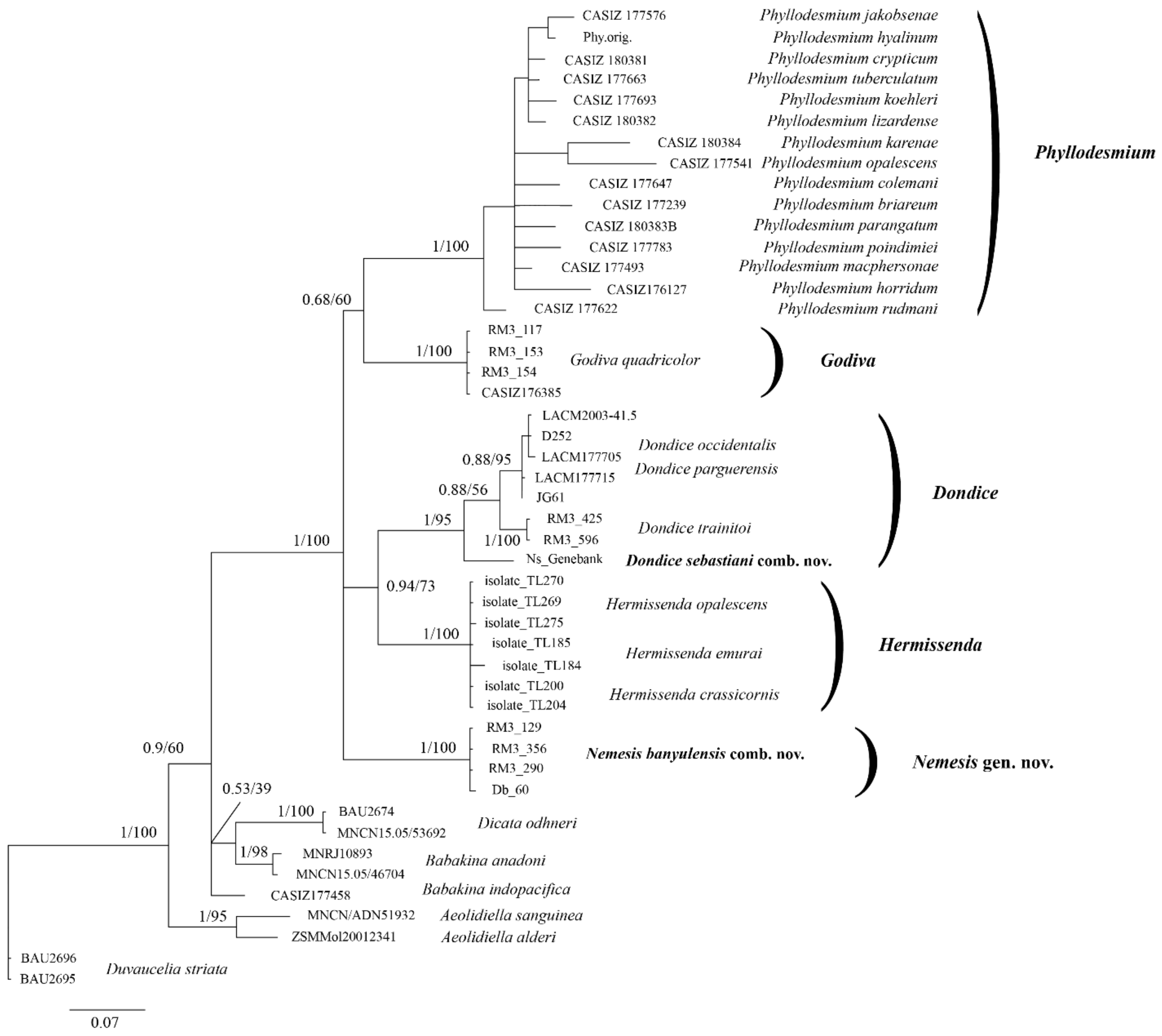

Supplementary Figure S1

Bayesian phylogenetic tree based on the single 16S dataset. Bayesian posterior probability (left) and Bootstrap (right) values are indicated at each node.

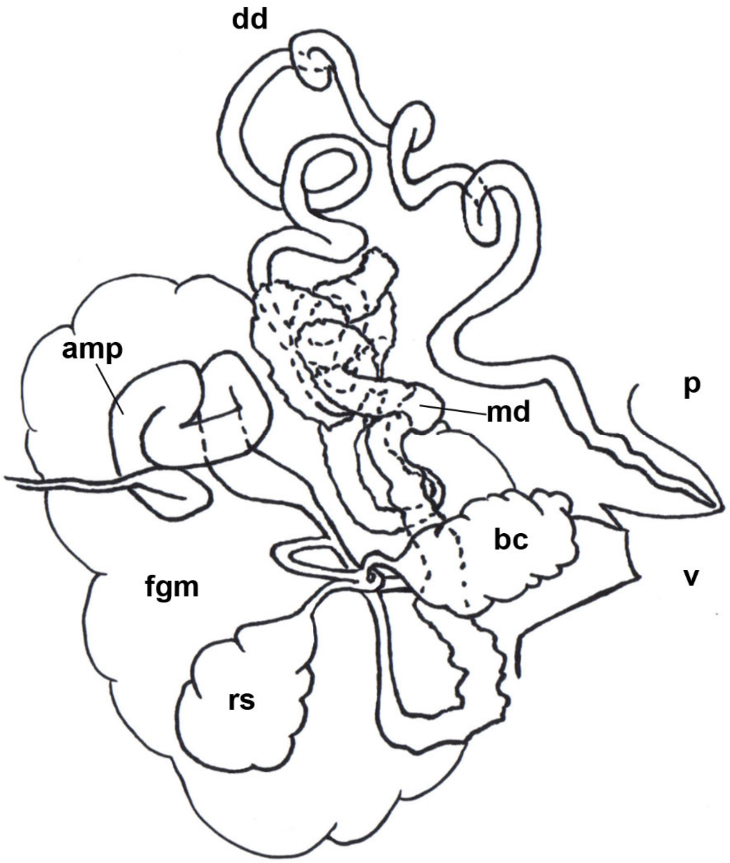

Supplementary Figure S2

Drawing of the reproductive system of *Nemesis banyulensis* comb. nov. (voucher RM3_290) performed using optical microscope. Legend: amp = ampulla, bc = bursa copulatory, dd = deferent duct, fgm = female gland mass, md = male duct, p = penis, rs = receptaculum seminis, v = vagina.

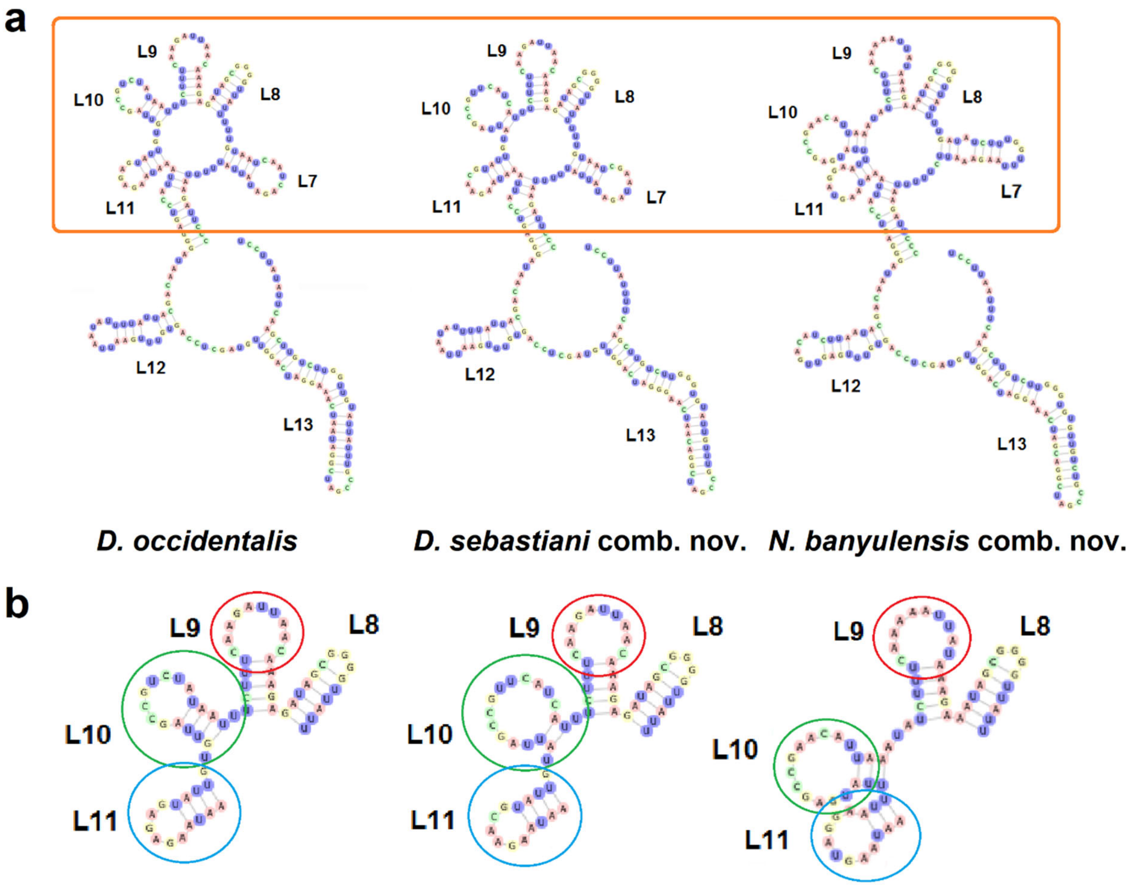

Figure S3: (a) Folding models of the 16S rRNA domain V of *D. occidentalis*, *D. sebastiani* comb. nov. and *N. banyulensis* comb. nov. analysed in the present work. The L7-11 stem-loops are boxed. (b) The variable L9-11 stem-loops are encircled to show differences occurring among the three taxa.

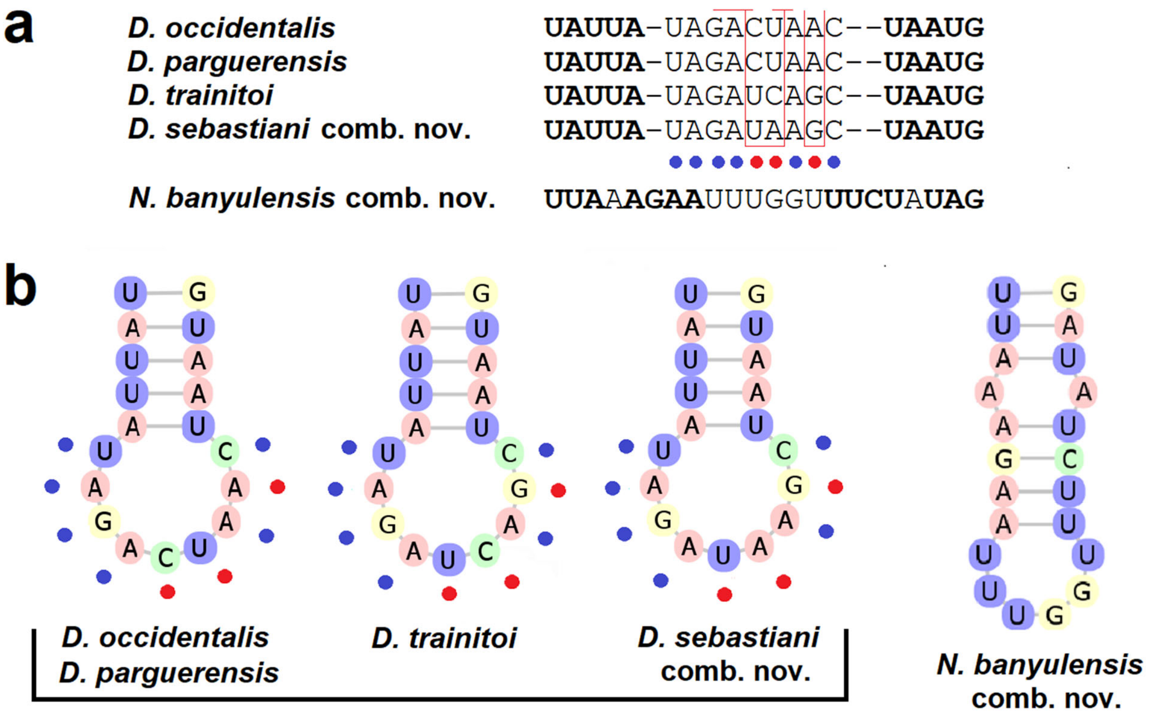

Figure S4: L7 stem-loops of the species here analysed. (a) Primary sequence alignment of the L7 stem-loops among the *Dondice* spp. and *N. banyulensis* comb. nov. (b) Folding models of the L7 stem-loops. Blue circles, conserved nucleotide positions within the *Dondice* clade, red circles diagnostic nucleotides.
